# Aberrant over-expression of a forkhead family member, *FOXO1A*, in a brain tumor cell line

**DOI:** 10.1186/1471-2407-7-67

**Published:** 2007-04-19

**Authors:** Peter B Dallas, Simone Egli, Philippa A Terry, Ursula R Kees

**Affiliations:** 1 Division of Children's Leukemia and Cancer Research, Telethon Institute for Child Health Research and Centre for Child Health Research, University of Western Australia, Perth, Australia

## Abstract

**Background:**

The mammalian FOXO (forkhead box, O subclass) proteins are a family of pleiotropic transcription factors involved in the regulation of a broad range of cellular processes critical for survival. Despite the essential and diverse roles of the FOXO family members in human cells and their involvement in tumor pathogenesis, the regulation of *FOXO *expression remains poorly understood. We have addressed the mechanisms underlying the high level of expression of the *FOXO1A *gene in a cell line, PER-453, derived from a primitive neuroectodermal tumor of the central nervous system (CNS-PNET).

**Methods:**

The status of the *FOXO1A *locus in the PER-453 CNS-PNET cell line was investigated by Southern blotting and DNA sequence analysis of the proximal promoter, 5'-UTR, open reading frame and 3'-UTR. FOXO1A expression was assessed by conventional and quantitative RT-PCR, Northern and Western blotting.

**Results:**

Quantitative real-time RT-PCR (qRT-PCR) data indicated that after normalization to *ACTB *mRNA levels, canonical *FOXO1A *mRNA expression in the PER-453 cell line was 124-fold higher than the average level of five other CNS-PNET cell lines tested, 24-fold higher than the level in whole fetal brain, and 3.5-fold higher than the level in fetal brain germinal matrix cells. No mutations within the *FOXO1A *open reading frame or gross rearrangements of the *FOXO1A *locus were detected. However, a single nucleotide change within the proximal promoter and several nucleotide changes within the 3'-UTR were identified. In addition, two novel *FOXO1A *transcripts were isolated that differ from the canonical transcript by alternative splicing within the 3'-UTR.

**Conclusion:**

The CNS-PNET cell line, PER-453, expresses *FOXO1A *at very high levels relative to most normal and cancer cells from a broad range of tissues. The *FOXO1A *open reading frame is wild type in the PER-453 cell line and the abnormally high *FOXO1A *mRNA expression is not due to mutations affecting the 5'-UTR or proximal promoter. Over expression of *FOXO1A *may be the result of PER-453 specific epimutations or imbalances in regulatory factors acting at the promoter and/or 3'-UTR.

## Background

The mammalian FOXO (forkhead box, O subclass) proteins, FOXO1A, FOXO3A, FOXO4, and FOXO6, are a family of transcription factors with complex and incompletely understood functional profiles [1-3]. Members of the family are involved in the regulation of a range of critical processes in mammalian cells, including proliferation, differentiation, apoptosis, metabolism, and responses to oxidative stress and DNA damage [4]. While some of these effects are due to reduced FOXO activity in the nucleus in response to signalling through the PI3K/Akt pathway [5], FOXO proteins integrate signals from multiple pathways and regulate gene expression as components of dynamic multi-protein complexes that vary with cell type and context [6]. This functional complexity is reflected both by the broad array of genes regulated by FOXO transcription factors and the diversity of post translational modifications regulating FOXO protein-protein interactions, intracellular location and degradation (for reviews see [7,8]).

In light of the pleiotropic nature of FOXO proteins and, in particular, the pivotal role of FOXO proteins as components of both the PI3K/Akt and TGFβ [9] pathways, both of which are frequently deregulated in cancer, it is not surprising that aberrant FOXO activity has been implicated in tumorigenesis [7]. Indeed, evidence is accumulating to suggest that the *FOXO *genes represent a tumour suppressor gene family [4]. In PTEN null prostate and glioblastoma cancer cell lines, reconstitution of nuclear FOXO1A or FOXO3A expression can suppress proliferation and induce senescence or apoptosis [10-12]. Data from the analyses of human primary tumor specimens have implicated the down regulation of FOXO1A expression in the pathogenesis of prostate [13] and endometrial cancer [14], as well as childhood alveolar rhabdomyosarcoma [15]. Although the molecular mechanisms of FOXO1A mediated tumor suppression are only partially understood it is likely that down regulation or FOXO1A expression potentiates tumorigenesis via deregulation of pathways that are context dependent. For example, reduced FOXO1A expression may contribute to the pathogenesis of glioblastoma through deregulation of TGFβ cytostatic signalling in neuroepithelial cells [9] while aberrant stoichiometry of FOXO1A-androgen receptor interactions may promote AKT-dependent and -independent survival of prostate cancer cells [13,16]. However, irrespective of cellular context, a complete understanding of the tumor suppressive properties of FOXO1A depends not only on the dissection of FOXO1A function at the protein level, but also the mechanisms of regulation of expression of *FOXO1A *mRNA.

The available data suggest that *FOXO1A *expression levels are generally low in primitive neuroectodermal tumours of the central nervous system (CNS-PNETs) [17]. However, our microarray expression analyses of CNS-PNET specimens revealed a surprisingly high level of *FOXO1A *expression in one CNS-PNET cell line relative to five other CNS-PNET cell lines and two normal fetal brain specimens. Although over expression of bona fide tumor suppressor genes such as *p16 *and *p53 *in cancer specimens has been reported [18,19] the molecular mechanisms by which this occurs and the biological significance of this phenomenon are poorly understood. Since *FOXO1A *is considered to be a tumor suppressor gene, and little is known about the regulation of *FOXO1A *mRNA expression levels in mammalian cells, we investigated the molecular mechanisms underlying the high expression of *FOXO1A *in the PER-453 CNS-PNET cell line.

## Methods

### Cell lines and control specimens

CNS-PNET cell culture conditions, and the origins and characteristics of the pineoblastoma cell lines PER-452, PER-453, and PER-480 have been described [20,21]. The medulloblastoma cell lines, PER-547 and PER-568 were established from biopsy specimens obtained from two boys, four and six years of age respectively, treated at Princess Margaret Hospital, Perth, Western Australia. The DAOY medulloblastoma cell line was obtained from Dr. Phillip Jacobsen, Royal Perth Hospital, Perth, Western Australia [22]. RNA was isolated from brain germinal matrix from a male fetus aborted at 16-weeks and fetal brain RNA (20 weeks gestation) was purchased (Clontech). Informed consent for the use of fetal tissues for research purposes was obtained for all individuals involved in this study according to hospital and Australian National Health and Medical Research (NHMRC) guidelines. The study was approved by the Princess Margaret Hospital Institutional Ethics Review Board.

### RNA extraction and microarray analysis

The procedure for RNA extraction, cRNA synthesis, and hybridisation to Affymetrix HG-U133A GeneChips has been described [23]. The microarray data were normalised using the Affymetrix MAS 5.0 software and recommended procedures.

### cDNA Synthesis, conventional RT-PCR, and DNA sequencing

cDNAs were synthesised from 2 μg total RNA using an oligo-dT primer and Omniscript RT kit (Qiagen) according to the manufacturer's protocol. Primers for conventional RT-PCR were designed using Primer Express™ 1.5 (PE Applied Biosystems) or MacVector™ 7.0r1 (Oxford Molecular). Details are available on request. Most RT-PCRs were performed with Taq Polymerase (Fisher Biotech) with the addition of 5% DMSO in some cases. The FailSafe PreMix Selection Kit (Epicentre) was employed to amplify the *FOXO1A *promoter and 5'-UTR which are particularly GC rich. A PTC-200 gradient cycler from GeneWorks was used for all PCR.

Amplified products were purified using either a PCR Clean Up (Qiagen) or Gel Extraction Kit (Qiagen). For cloning we used a TOPO Cloning Kit (Invitrogen). PCR products or plasmid clones were sequenced using Big Dye Terminator V3, and the Applied Biosystems (ABI) PRISM 3730 capillary sequencer.

### Quantitative RT-PCR (qRT-PCR)

qRT-PCR assays were carried out using a primer and probe set specific for human *FOXO1A *(ABI, Assay on Demand). Aliquots of total RNA extracted for microarray analysis as described above were used for qRT-PCR experiments according to ABI protocols. qRT-PCRs were run on an ABI 7700 sequence detector. All qRT-PCR experiments were carried out in duplicate and expression levels were normalized to *ACTB *levels.

### Northern blot analysis

PolyA+ mRNA was isolated using a Qiagen Oligotext mRNA Mini Kit. mRNA (2 ug) was prepared with deionized glyoxal, electrophoresed on a 1.2% agarose gel and transferred in 20×SSC to Magna Neutral Membrane (Osmonics Inc.). The membrane was probed with a gel purified ^32^P labelled *FOXO1A *PCR product and autoradiographed. The membrane was stripped and re-probed with a ^32^P labelled *ACTB *cDNA probe as a loading control.

### Immunoblots

#### Whole cell extract

Whole cell protein extracts were obtained from cell lines PER-452, PER-453 and PER-480 using lysis buffer containing 2 mM EDTA, 1.85 mg/ml iodoacetamide, 25 μg/ml p-nitrophenylguanidinobenzoate, 10 μg/ml leupeptin, 10 μg/ml aprotinin and 0.5% Triton X-100. Protein concentrations were determined using BioRad Protein Assay reagent. Total protein (20 μg) was electrophoresed on a 10% SDS PAGE gel and transferred to Hybond-C super membrane (Amersham). The membrane was blocked in 0.1% TBST/5% skim milk for 1 hr at RT and incubated overnight at 4°C with FOXO1A antibody (Cat. No. 9462, Cell Signaling Technology). Following washing, the membrane was incubated with anti-Rabbit HRP (Amersham) for 2 hr at RT. The membrane was washed, treated with ECL reagent (Amersham) for one minute and exposed to ECL film (Amersham).

### Nuclear and cytoplasmic extracts

Nuclear and cytoplasmic protein fractions were obtained from the PER-453 cell line. Cells were resuspended in 0.5 ml of Buffer A (10 mM Hepes, 10 mM KCl, 0.1 mM EDTA, 0.1 mM EGTA, 1 mM DTT, 10 μg/ml aprotinin, 10 μg/ml leupeptin, and 0.1 mM sodium vanadate), mixed gently and incubated on ice for 15 minutes. The samples were vortexed after addition of 55 μl 10% Nonidet-P-40, centrifuged for one minute at 4°C, and the supernatants collected. The remaining pellets were resuspended in 100 μl ice cold Buffer B (20 mM Hepes, 400 mM NaCl, 1 mM EDTA, 1 mM EGTA, 1 mM DTT, 10 μg/ml aprotinin, 10 μg/ml leupeptin, and 0.1 mM sodium vanadate), rocked for ten minutes at 4°C, centrifuged, and the nuclear protein containing supernatant was collected and analyzed (see above). To control for fractionation, the membrane was re-blocked in TBST/10% skim milk, incubated with antibodies to ACTB (Pan-Actin Ab-5, Neomarkers) or Histone H1 (AE4, Santa Cruz) at RT for 2 hr, washed and incubated with anti-Mouse HRP (Amersham). The membrane was washed, treated with ECL reagent, and exposed to ECL film (see above). Chemiluminescence was assessed by phosphoimagery (Fujifilm FLA-3000 phosphoimager) and quantitation by densitometry was undertaken using Fujifilm software (Image Gauge Version 4.22).

### Southern blot analysis

Genomic DNA was extracted from cell lines using standard procedures and from peripheral blood lymphocytes (PBLs) using the QIAamp DNA Blood Mini Kit (Qiagen). DNA (15 μg) was digested with *Eco*R1 (New England BioLabs) at 37°C, electrophoresed overnight on a 0.8% agarose gel, and transferred to Hybond-N+ membrane (Amersham) using 0.4 M NaOH. The membrane was hybridized with the same gel purified ^32^P labelled cDNA probe used for Northern analysis. To control for DNA loading, the blot was stripped and re-probed with a chromosome 14 specific genomic PCR product expected to be present at normal diploid levels in the PER-453 cell line. Hybridization signals were obtained by phosphoimagery and densitometric analysis was undertaken as described above for immunoblotting.

### Luciferase assays

Wild-type and mutant genomic PCR products spanning 1161 bp of sequence from -866 to +296 relative to the *FOXO1A *transcription start site were generated from PER-453 and cloned into the luciferase vector, pGL3-basic (Promega). Human embryonic kidney (HEK) cells were transfected simultaneously with 400 ng of test plasmid and β-galactosidase expressing transfection control plasmid pSV-β gal (Promega), using Lipofectamine 2000 according to the manufacturer's protocol (Invitrogen). After 48 hours, luciferase and b-galactosidase activities were assayed using the β-galactosidase enzyme assay system with reporter lysis buffer (Promega) according to the manufacturer's instructions. Assays were carried out in duplicate and measurements of normalised luciferase activity were obtained from two independent experiments.

## Results

### Expression of FOXO1A mRNA and protein in CNS-PNET cell lines

**Figure 1:**
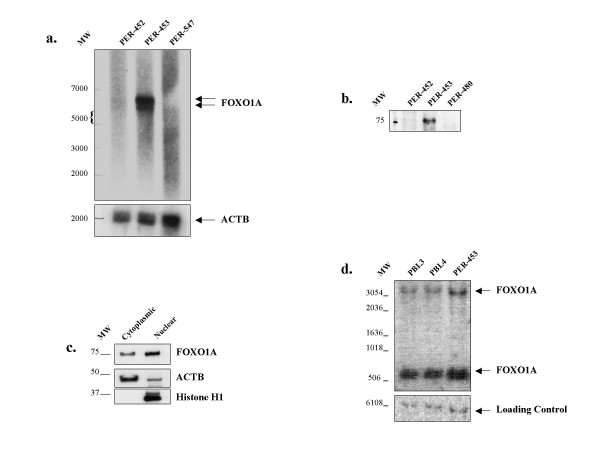
**a) *FOXO1A *Northern analysis**. mRNA isolated from the PER-452, PER-453, and PER-547 CNS-PNET cell lines was screened with a ^32^P labelled *FOXO1A *RT-PCR product spanning *FOXO1A *exons 1 and 2 including part of the forkhead domain (see Fig. 2b). A prominent band of ~5.9 kb, and a weaker band of ~5.4 kb were clearly visible in the PER-453 lane and are indicated by arrows on the upper right. Faint bands of similar sizes were visible in the PER-452 lane on the original autoradiograph and no signals in this size range were observed in the PER-547 lane. Molecular weight markers (nt) are included on the left. **b**) **FOXO1A immunoblot**. Total cell lysates from the PER-452, PER-453, and PER-480 CNS-PNET cell lines were screened with an antibody to FOXO1A. **c) **A ~78 kDa band, equivalent to the known size of wild type FOXO1A, is clearly visible in the PER-453 lane. A weak signal at 78 kDa was also detected in the PER-452 lane, and no signal was detected in the PER-480 lane. Equal proportions (~7%) of either the nuclear or cytoplasmic protein fractions isolated from the PER-453 cell line were also assessed with the FOXO1A antibody. FOXO1A protein was detected in both fractions with a greater proportion detectable in the nucleus. As fractionation controls the blot was re-probed with monoclonal antibodies to the predominantly cytoplasmic protein ACTB, and nuclear protein histone H1. Molecular weight markers (kDa) are included on the left. **d**) ***FOXO1A *gene copy number analysis**. Genomic DNA from the PER-453 cell line and two normal individuals was digested with *Eco*RI and analysed by Southern analysis using a *FOXO1A *specific cDNA probe. *FOXO1A *genomic fragments of 3320 bp and 613 bp are indicated by arrows in the top panel. To control for loading the blot was stripped and reprobed with a ^32^P labelled PCR product mapping to chromosome 14q that was expected to be present at diploid levels in the three specimens (bottom panel).

We assessed the transcription profiles of five CNS-PNET cell lines, whole human fetal brain (20 weeks), and human fetal brain germinal matrix cells (16 weeks) using Affymetrix HG-U133A microarrays. Analysis of the microarray data revealed a very high level of *FOXO1A *mRNA in the PER-453 cell line relative to the other CNS-PNET cell lines and normal fetal brain specimens (Table 1). Verification of the microarray data using quantitative real-time RT-PCR (qRT-PCR) indicated that after normalization to *ACTB *mRNA levels, canonical *FOXO1A *mRNA expression in the PER-453 cell line was 124-fold higher than the average level of the other five CNS-PNET cell lines, 24-fold higher than the level in whole fetal brain, and 3.5-fold higher than the level in fetal brain germinal matrix cells (Table 1). We also assessed *FOXO1A *mRNA expression in the PER-452, PER-453, and PER-547 CNS-CNS-PNET cell lines by Northern blotting (Fig. 1a). A prominent signal at ~5.9 kb, consistent with the size of the wild type *FOXO1A *transcript [24], and a lesser signal at ~5.4 kb were visible in the PER-453 lane, while very faint signals of ~5.9 kb and ~5.4 kb were visible on the original autoradiograph for PER-452. *FOXO1A *mRNA was undetectable in the PER-547 cell line consistent with the qRT-PCR results. *ACTB *hybridisation signals indicated that an adequate amount of mRNA was loaded in each lane.

To assess FOXO1A protein levels in CNS-PNET cell lines PER-452, PER-453, and PER-480, whole cell lysates were analyzed by immunoblotting (Fig. 1b). FOXO1A protein was weakly detectable in the PER-452 cell line and was prominent in the PER-453 cell line at approximately 78 kDa. These data are consistent with the qRT-PCR results and the expected molecular weight of the wild type FOXO1A protein. No signal was observed in the PER-480 cell line consistent with undetectable *FOXO1A *mRNA levels in this cell line as measured by qRT-PCR.

FOXO1A is a transcription factor that is known to shuttle between the nucleus where it is functionally active, and the cytoplasm where it is considered to be functionally inactive, in response to changes in its phosphorylation status. To assess the subcellular localisation of FOXO1A in the PER-453 cell line we investigated nuclear and cytoplasmic fractions by immunoblotting. Screening with control antibodies against the predominantly cytoplasmic protein ACTB, and the nuclear histone protein H1, indicated that the nuclear and cytoplasmic fractionation was successful. Quantitative densitometry revealed that FOXO1A protein levels were approximately 2.85 fold higher in the nuclear fraction than in the cytoplasm (Fig. 1c).

### Genetic analysis of the FOXO1A locus in the PER-453 cell line

**Figure 2:**
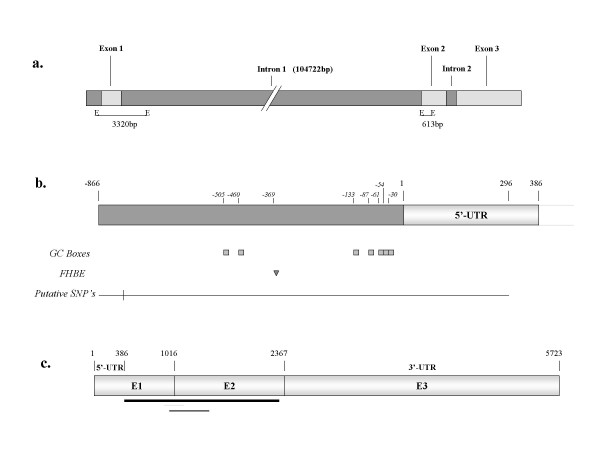
**a) The human *FOXO1A *locus**. Genomic arrangement of the *FOXO1A *locus on chromosome 13q14.11, and position and sizes (3320 bp and 613 bp) of known *Eco*RI (E) restriction sites. **b) Analysis of the PER-453 *FOXO1A *proximal promoter**. The positions of seven putative GC-boxes (shaded squares), an FHBE (shaded triangle), and the single G to A base change identified in the *FOXO1A *promoter are indicated (see Table 3 for more details). **c) Schematic depiction of the *FOXO1A *mRNA**. The *FOXO1A *open reading frame of 1965 bp (thick line), forkhead DNA binding domain (dotted line), and the location of the cDNA probe used for Northern and Southern analysis (thin line) are indicated below the map.

To investigate the underlying mechanism for the high expression of *FOXO1A *in the PER-453 cell line we carried out a detailed analysis of the *FOXO1A *locus and cDNA sequence. Classical cytogenetic analysis of the PER-453 genome has been reported previously [20] and recently high-resolution array-CGH analysis of the PER-453 cell line was completed. All metaphase spreads examined were near diploid and copy number variations or gross chromosomal rearrangements likely to affect the *FOXO1A *gene on chromosome 13 were not detected using either technique. A common mechanism associated with over-expression of specific genes in cancer (generally oncogenes), is gene amplification. Accordingly, we assessed whether over-expression of *FOXO1A *in the PER-453 cell line was associated with increased *FOXO1A *gene copy number that may not have been detected by cytogenetic analysis. Genomic DNAs from the PER-453 cell line and PBLs from two normal individuals were digested with *Eco*RI and probed with a *FOXO1A *cDNA probe. Hybridization to two *Eco*RI restriction fragments of 3320 bp and 613 bp was observed as expected (Figs 1d and 2a). Although there was a higher DNA loading in the PER-453 lane relative to the PBL control lanes as judged by the relative signals of the loading control probe, densitometry followed by linear regression analyses revealed that there were no significant differences between the ratios of *FOXO1A *hybridisation signal to control signals (p = 0.29)(Table 2). These data indicate that *FOXO1A *gene copy number is not increased in the PER-453 cell line.

### Sequence analysis of the FOXO1A gene

We hypothesised that the high *FOXO1A *mRNA expression in PER-453 cells may be the result of aberrant transcriptional regulation due to mutations or rearrangements in sequences upstream of the *FOXO1A *open reading frame or within the 3'-UTR. Schematic representations of the organization of the *FOXO1A *locus at 13q14.11 and the canonical *FOXO1A *cDNA sequence (GenBank accession no. NM_002015; [24]) are presented in Fig 2. We generated a series of overlapping RT-PCR products covering 96% of the *FOXO1A *cDNA from PER-453. In addition, two overlapping genomic PCR products were generated spanning the *FOXO1A *proximal promoter.

### Identification of a single nucleotide change in the FOXO1A promoter

Genomic PCR products spanning 1161 bp of sequence from -866 to +296 relative to the *FOXO1A *transcription start site were generated from PER-453 and five other CNS-PNET cell lines, three of which do not express *FOXO1A*. All DNA sequences were identical except for a single nucleotide difference (A to G) detected at position -832 of one *FOXO1A *allele in the PER-453 cell line (Fig. 2b and Table 3).

We analysed the proximal promoter sequences 866 bp upstream of the major *FOXO1A *transcription start site [25] for transcription factor binding sites using MatInspector [26]. Seven putative GC-boxes (GGGCGG) were detected which may represent binding sites for transcriptional regulators such as the specificity protein family members, SP1-SP4 [27]. We also identified a consensus forkhead-binding element (FHBE) (GTAAACAAA) [28] in the *FOXO1A *proximal promoter at position -371 to -363 relative to the *FOXO1A *transcription start site (Fig. 2).

### Identification of five single nucleotide changes and two splice variants in the FOXO1A 3'-UTR

**Figure 3:**
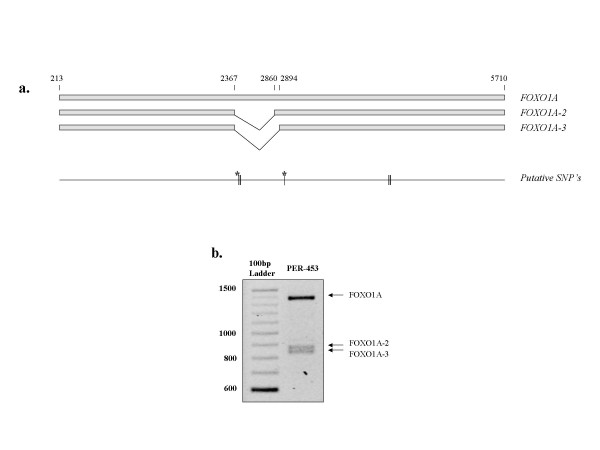
**a) Summary of *FOXO1A *cDNA sequencing and analysis**. The structures of two *FOXO1A *splice variants (*FOXO1A-2 *and *FOXO1A-3*) are indicated. Two previously identified (*), and three additional single nucleotide differences were identified (see Table 3 for more details). **b**) **Identification of alternative *FOXO1A *transcripts**. RT-PCR analysis of the PER-453 cell line using *FOXO1A *specific primers (see Fig. 2b) generated amplicons of 1364 bp (canonical *FOXO1A*), 872 bp (*FOXO1A-2*), and 838 bp (*FOXO1A-3*). RT-PCR products were electrophoresed on 0.8% ethidium bromide stained agarose gels.

**Figure 4:**
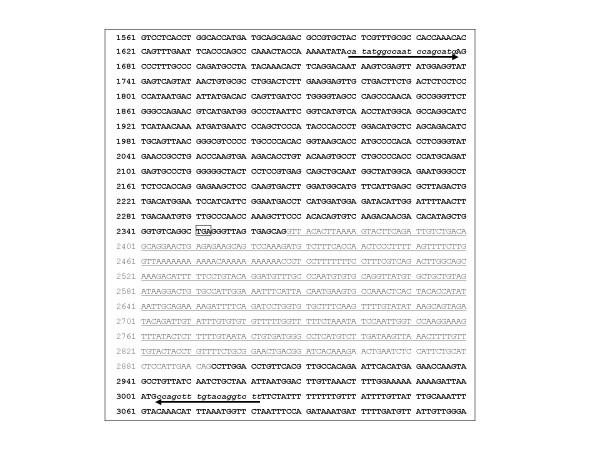
**DNA sequence of the alternatively spliced region of the *FOXO1A *3'-UTR**. The alternatively spliced regions of the *FOXO1A-2 *and *FOXO1A-3 *variants are in plain text. DNA sequence flanking the spliced region is in bold. The alternatively spliced region of the *FOXO1A-2 *transcript is underlined and is 34 bp shorter than the region spliced from the *FOXO1A-3 *transcript. The *FOXO1A *stop codon is boxed. Primers used to amplify the alternatively spliced region are in italics and underlined with an arrow.

The *FOXO1A *mRNA has a particularly long 3'-UTR of 3356 nt representing nearly 60% of the length of the transcript (Fig. 2c). The extent of the *FOXO1A *3'-UTR suggests that this region may play an important role in the regulation of *FOXO1A *transcript stability or translation. We did not identify any gross rearrangements in the *FOXO1A *3'-UTR from PER-453 by DNA sequence analysis. However, we identified five single nucleotide differences including two previously identified SNPs (Table 3 and Fig. 3a). In addition to the canonical cDNA sequence, we identified two *FOXO1A *splice variants designated *FOXO1A-2 *and *FOXO1A-3*, both of which are differentially spliced in a manner consistent with the GT/AG rule from the same splice donor site as the canonical transcript, 14 bp downstream from the stop codon within the 3'-UTR (Fig. 3b and Fig 4). The sequences of these cDNAs were identical to the canonical *FOXO1A *cDNA except for the splicing out of 492 bp (extending from nt 2368 – 2859, *FOXO1A-2*) and 526 bp (extending from nt 2368–2893, *FOXO1A-3*)(Fig. 4). We did not obtain evidence for any other *FOXO1A *alternative splicing events from any of our RT-PCR analyses, which also included amplification of the entire *FOXO1A *cDNA as two overlapping RT-PCR products. These data indicated that the *FOXO1A-2 *and *FOXO1A-*3 cDNAs were derived from transcripts that were identical to the canonical transcript except for the alternative splicing within the 3'-UTR. The lower signal intensity and size of the ~5.4 kb molecular weight band relative to the canonical ~5.9 kb transcript visible on the Northern blot (Fig. 1a) was consistent both with the expected sizes of the smaller alternative transcripts and the levels of the RT-PCR products generated from the two splice variants relative to the canonical *FOXO1A *RT-PCR product when observed on ethidium bromide stained agarose gels (Fig. 3b).

### Comparison of FOXO1A wild-type and mutant promoter activity

Luciferase assays were employed to assess the affect on expression of the single A to G base change identified in one allele of the *FOXO1A *promoter in the PER-453 cell line. Linear regression analysis of the data from two independent experiments carried out in duplicate indicated that the single nucleotide change had no significant affect on promoter activity (p = 0.8).

## Discussion

Despite the essential and diverse roles of the FOXO family members in human cells, the regulation of FOXO expression is poorly understood. FOXO proteins are important components of signalling nodes responsive to external cellular cues, and compromised FOXO function and consequent disruption of these nodes is strongly implicated in tumorigenesis [7,9,29]. Most studies of the role of FOXO proteins in carcinogenesis have concentrated on the regulation of FOXO activity at the post-translational level, especially with regard to nucleo-cytoplasmic shuttling in response to changes in FOXO phosphorylation status. However, FOXO expression levels and the regulation of *FOXO *transcription have received scant attention in any context.

According to the microarray data the normalised *FOXO1A *expression level (probeset 202724_s_at) in the PER-453 cell line was 5.4-fold higher than the *FOXO1A *level of normal fetal brain. Furthermore, it was 4-fold higher than the median expression level of *FOXO1A *in 73 different human tissues (including normal fetal brain) and cell lines that were assessed by others using HG-U133 GeneChips [30]. Similarly, *FOXO1A *was generally expressed at low levels in the 83 cancer specimens assessed by Su *et al*. These data suggest that the expression level of *FOXO1A *in the PER-453 cell line is exceptionally high relative to the levels in most normal and cancerous tissues. We were unable to assess *FOXO1A *expression levels in the primary pineoblastoma from which the PER-453 cell line was derived. However, the primary specimen and cell line displayed very similar immunophenotypes and ultrastructural characteristics [20] suggesting that the cell line has not diverged greatly from that of the primary tumor. Although there have been no published studies specifically addressing *FOXO1A *expression levels in CNS-PNETs, the vast majority of primary CNS-PNET specimens assayed using the superseded HG-UL95A GeneChip were called "absent" for *FOXO1A *expression in the largest published microarray study of 68 CNS-PNET specimens [17], suggesting that *FOXO1A *expression is generally low in primary CNS-PNETs.

Chromosomal translocations involving *FOXO *family members have been described in human leukemias and pediatric alveolar rhabdomyosarcomas [31]. In each case, FOXO fusion proteins result from translocations generating chimeric transcripts encoding the N-terminal region of the fusion partner (*MLL *for the leukemias, and either *PAX3 *or *PAX7 *for the rhabdomyosarcomas), and the C-terminal portion of the *FOXO *component. Hence, the expression levels of the chimeric transcripts are presumably regulated through the 5' promoter sequences of the various *FOXO *fusion partners in combination with the *FOXO *3'-UTR. These findings raise the possibility that the *FOXO1A *over-expression observed in the PER-453 cell line could be the result of a fusion event. However, our data suggest that this is unlikely. Immunoblotting analysis of the PER-453 cell line using a FOXO1A antibody that recognizes an epitope C-terminal to the usual FOXO1A fusion point revealed a prominent band of approximately ~78 kDa. This is consistent with the expected size of wild type FOXO1A and is significantly smaller than FOXO1A-PAX3/PAX7 (~97 kDa)[32], or FOXO-MLL (>200 kDa) fusion proteins [33].

The lack of coding region mutations or rearrangements, and the predominantly nuclear localization of the FOXO1A protein are consistent with functionally normal FOXO1A protein in PER-453 cells. In addition, our data indicated that *FOXO1A *over-expression in the PER-453 cell line was not associated with gene amplification or a translocation event and we did not detect gross rearrangements of the *FOXO1A *proximal promoter, 5'-UTR, or 3'-UTR. These results suggest that *FOXO1*A over-expression may be the result of altered chromatin structure (e.g. histone hyperacetylation) at the *FOXO1A *locus and/or aberrant regulation by factors acting in trans at the promoter and/or 3'-UTR. The expression levels of several genes flanking the *FOXO1A *locus were similar in each of the CNS-PNET cell lines, suggesting that a generalised chromatin restructuring and/or coordinated deregulation of genes in the region of 13q14.11 is unlikely to explain the aberrant *FOXO1A *expression observed in the PER-453 cell line. To date, there have been no detailed structural or functional analyses of the promoters or 3'-UTRs of any member of the FOXO family. Although the extent of the human *FOXO1A *promoter has yet to be formally determined, our data confirm that the proximal promoter is TATA-less and GC rich [25]. Genes with promoters of this kind are often regulated, at least in part, by the trans acting specificity factors SP1-SP4 [34]. In addition, the identification of a FHBE in the *FOXO1A *promoter raises the possibility that forkhead transcription factors may be involved in the regulation of *FOXO1A *message levels. A more detailed analysis is required to determine whether imbalances in SP and/or forkhead transcription factors are associated with deregulated *FOXO1A *expression in the PER-453 cell line.

The luciferase data suggest that the A to G mutation identified in one allele of the FOXO1A promoter in the PER-453 cell line is not associated with *FOXO1A *over-expression. Although we were unable to test the activities of the luciferase reporter constructs in CNS-PNET cells because of the low transfection efficiency of these cells, we considered that HEK cells represented a reasonable compromise for our study because they are tumorigenic cells derived from human foetal tissue and they express *FOXO1A *mRNA at a level comparable to human foetal brain cells, from which CNS-PNETs are thought to be derived. Overall, our data suggest that the high *FOXO1A *expression in the PER-453 cell line is associated with imbalances in transcription factor activity or epigenetic changes that are specific to this cell line.

It is becoming increasingly clear that the 3'-UTRs of eukaryotic transcripts can play important roles in the regulation of mRNA stability, localisation, and translation [35], and aberrant 3'-UTR mediated regulation is involved in oncogenesis [36]. We have identified two novel *FOXO1A *transcripts in the PER-453 cell line that differ from the canonical transcript through splicing out in their 3'-UTRs. Apart from the report of a low abundance alternative *FOXO1A *transcript isolated from a human fetal brain cDNA library and distinct from those described here [24], this is the first demonstration of other alternatively spliced *FOXO1A *transcripts in any cell type. It remains to be seen whether these alternative *FOXO1A *transcripts are brain tissue specific or present in other cell types, and whether the alternatively spliced region is involved in the regulation of transcript abundance and tissue specific expression. A detailed analysis of the significance of *FOXO1A-2 *and *FOXO1A-3 *expression in human cells may provide new insights into the mechanisms by which FOXO1A expression levels are finely coordinated in response to diverse stimuli.

Although some of the specific 3'-UTR sequences and trans-acting factors that are involved in these processes have been determined, the precise mechanisms of 3'-UTR mediated regulation of transcript abundance remain poorly understood. An analysis of the canonical *FOXO1A *3'-UTR sequence for known 3'-UTR regulatory elements by UTRscan [37] revealed only a consensus Brd-box (AGCTTA) and K-box (TGTGAT). These repressive elements, first described in the 3'-UTRs of multiple genes of the *enhancer of split *complex in *Drosophila*, are thought to be involved in miRNA mediated regulation of Notch signalling [38]. Interestingly, one of these elements, the K-box, is not present in either of the alternatively spliced *FOXO1A *transcripts that we detected in PER-453 cells. At present, it is unknown whether mammalian Brd-box and K-box elements are functionally similar to their insect counterparts, and it remains to be determined whether these elements play a role in miRNA-mediated regulation of *FOXO1A *expression.

Although apparently paradoxical, high expression of bona fide tumor suppressor genes, including *p16 *[18] and *p53 *[19], have been reported in solid tumor specimens. In the latter study, high levels of wild type p53 were detected in CNS-PNETs. These authors suggest that apoptosis that would normally be induced by high p53 levels is counterbalanced by elevated expression of anti-apoptotic Bcl-2 in these tumors. It is possible that an analogous situation exists in the PER-453 cell line whereby the tumor suppressive effects of the high level of expression of wild type FOXO1A are overridden by inactivation or deregulation of downstream effector molecules.

## Conclusion

The importance of FOXO family members as critical components of signalling nodes implicated in the pathogenesis of a variety of tumor types suggests that the modulation of FOXO expression and function may be a useful approach for the treatment of some human cancers [39,40]. On that basis, a greater understanding of FOXO transcriptional regulation, as well as FOXO protein shuttling dynamics and post-translational modification, may well provide new avenues for the design of novel therapies targeting FOXO activity in cancer cells.

The CNS-PNET cell line, PER-453, expresses *FOXO1A *at very high levels relative to most normal and cancer cells from a broad range of tissues. The *FOXO1A *open reading frame is wild type in the PER-453 cell line and the abnormally high *FOXO1A *mRNA expression is not due to mutations affecting the 5'-UTR or proximal promoter. Over expression of *FOXO1A *may be the result of PER-453 specific epimutations or imbalances in regulatory factors acting at the promoter and/or 3'-UTR.

## Competing interests

The author(s) declare that they have no competing interests.

## Authors' contributions

PBD designed the study, analysed and interpreted the data, and wrote the manuscript. SE and PAT carried out the experiments and contributed to the interpretation of data and drafting of the manuscript. URK was involved in study design, data interpretation and manuscript preparation. All authors read and approved the final manuscript.

## Pre-publication history

The pre-publication history for this paper can be accessed here:



## Figures and Tables

**Table 1:** *FOXO1A *mRNA expression levels in CNS-PNET cell lines and normal fetal brain specimens.

	**EXPRESSION OF *FOXO1A***		
**Cell line/tissue**	**MICROARRAY**	**qRT-PCR**	**N qRT-PCR**

PER-452	228	0.57	0.65
**PER-453**	**2324**	**19.89**	23.86
PER-480	A	0.009	0.01
PER-547	A	0.006	0.01
PER-568	A	0.005	0.01
DAOY	ND	0.21	0.24
FETAL BRAIN (FB)	428	0.87	1
GERMINAL MATRIX	887	5.9	6.78

*FOXO1A *mRNA expression levels based on microarray expression scores were obtained for CNS-PNET cell lines and controls using HG-U133A GeneChips (202724-s-at probe set, 3'-UTR specific). Relative expression levels were verified by qRT-PCR and are expressed relative to *ACTB *expression levels in each specimen. Normalised qRT-PCR (NqRT-PCR) levels are presented relative to fetal brain. A-absent call, ND-not determined.

**Table 2:** Densitometric analysis of the *FOXO1A *Southern blot hybridization signals in Fig. 1d.

	**Ratio FOXO1A/loading control**	
	
**DNA source**	**613 bp signal**	**3320 bp signal**
**PER-453**	1.32	1.13
**PBL-3**	1.46	1.27
**PBL-4**	1.37	1.12

**Table 3:** Nucleotide differences in the *FOXO1A *3'-UTR.

**DNA source**	**Position of Base Change Relative to Transcriptional Start Site**	**Base Change Observed**	**Published SNP NCBI – SNP Database Build 126**
**Promoter Region Clones**	-832	A/G	**No**
**PCR Product**	2464 to 2474	single base insertion (A)	Yes dbSNP: 11406284
**PCR Product and Clone**	2476 to 2486	single base deletion (A)	No
**PCR Product and Clone**	2986 to 2994	single base deletion (A)	Yes dbSNP: 3841231
**PCR Product**	4338	G/C	No
	4356	C/T	No

Overlapping genomic or RT-PCR fragments were sequenced and compared to the canonical *FOXO1A *sequence (Genbank accession no. NM_002015).
